# Phenology and Floret Development as Affected by the Interaction between *Eps-7D* and *Ppd-D1*

**DOI:** 10.3390/plants10030533

**Published:** 2021-03-12

**Authors:** Priyanka A. Basavaraddi, Roxana Savin, Stefano Bencivenga, Simon Griffiths, Gustavo A. Slafer

**Affiliations:** 1 Department of Crop and Forest Sciences-AGROTECNIO CERCA Center, University of Lleida, Av. R. Roure 191, 25198 Lleida, Spain; priyanka.basavaraddi@udl.cat (P.A.B.); roxana.savin@udl.cat (R.S.); 2 John Innes Centre, Norwich Research Park, Colney Ln, Norwich NR4 7UH, UK; stefano.bencivenga@gmail.com (S.B.); simon.griffiths@jic.ac.uk (S.G.); 3 ICREA, Catalonian Institution for Research and Advanced Studies, 23, 08010 Barcelona, Spain

**Keywords:** spike fertility, leaf appearance, spikelet primordia, earliness per se, photoperiod

## Abstract

Earliness per se (*Eps*) genes may play a critical role in further improving wheat adaptation and fine-tuning wheat development to cope with climate change. There are only few studies on the detailed effect of *Eps* on wheat development and fewer on the interaction of *Eps* with the environment and other genes determining time to anthesis. Furthermore, it seems relevant to study every newly discovered *Eps* gene and its probable interactions as the mechanisms and detailed effects of each *Eps* may be quite different. In the present study, we evaluated NILs differing in the recently identified *Eps-7D* as well as in *Ppd-D1* at three temperature regimes (9, 15 and 18 °C) under short day. The effect of *Eps-7D* on time to anthesis as well as on its component phases varied both qualitatively and quantitatively depending on the allelic status of *Ppd-D1* and temperature, being larger in a photoperiod-sensitive background. A more noticeable effect of *Eps-7D* (when combined with *Ppd-D1b*) was realised during the late reproductive phase. Consequently, the final leaf number was not clearly altered by *Eps-7D*, while floret development of the labile florets (florets 2 and 3 in this case, depending on the particular spikelet) was favoured by the action of the *Eps-7D-late* allele, increasing the likelihood of particular florets to become fertile, and consequently, improving spike fertility when combined with *Ppd-D1b*.

## 1. Introduction

As mentioned in the introduction of the companion paper [1], and elsewhere [2,3], the main genetic factors controlling wheat developmental traits are photoperiod- and vernalisation-sensitivity genes (*Ppd* and *Vrn*), both critical for coarse tuning phenology, and the earliness per se (*Eps*) gene, responsible for fine-tuning development [4]. Furthermore, these genetic factors controlling the duration of the developmental phases may have pleiotropic effects on yield traits [5,6], explaining a selective advantage of certain genes over others. Yield advantages of these genetic factors may depend on the strength of their effect on phenology and whether they also influence the rate and development of organs/primordia along the way. A number of studies showed that genes lengthening the duration of either time until terminal spikelet (TS) or the late reproductive phase (LRP, from then to anthesis) may affect the number of organs initiated during these phases [7,8,9,10,11].

The importance and application of genetic factors controlling developmental traits may depend on the epistatic interaction with one another and, in turn, with environmental conditions. In spring wheat, only the *Ppd* and *Eps* genes can be manipulated to alter patterns of development and associated traits. *Eps* genes are a group of completely different genes spread over the genome [2], only having in common what defines them: producing a relatively minor effect on time to heading/anthesis when requirements of photoperiod and vernalisation are fully met [12]. At least in part, the ambiguity of the results reported in the literature may well reflect the fact that the mechanism of each *Eps* gene could be quite different from one another, beyond the fact that all affect time to heading/anthesis (by definition). Final leaf number (FLN), phyllochron, plastochron, spikelet number, floret development patterns and developmental phases can be affected or not depending on the specific *Eps* gene studied [7,8,9,13,14,15,16]. Therefore, it is important to study and evaluate each novel *Eps* along with their interactions with the other genetic factors (involved in complex network of genes altering the life cycle of wheat) and the environment. The former interaction would identify the kind of genetic background in to which a particular *Eps* can be introgressed, resulting in optimum function. Determining to what degree the environmental condition affect the effects on phenology of a particular *Eps* and its interaction with the background is important if we are to predict the environment in which such a genetic combination can confer maximum benefit. For instance, in the companion paper, we showed that the environment affected the magnitude of effects of a novel *Eps* gene (*Eps-7D*) on phenology and spike fertility traits [1]; moreover, in a recent paper [16], we showed that these effects of *Eps-7D* were affected by its interaction with another *Eps* gene (*Eps-2B*). Quantifying the interactions with major genes coarse-tuning wheat development is relevant to determine whether it is potentially useful to exploit a particular *Eps* gene in a certain wheat background.

The interaction between *Eps* and *Ppd* genes was evaluated in a previous study, which showed that the two genes have an additive interaction [17]. Similarly, another recent study has shown differential effect of *Eps* on heading time in presence of either *Ppd* sensitive or insensitive alleles [13]. These studies suggest the importance of understanding not just the independent effects of any new *Eps* gene discovered but also its interaction with *Ppd* alleles. The outcome of the above-mentioned studies is limited to the *Eps* × *Ppd* interaction effect on time to heading/anthesis. Such interactions were not studied yet for the recently identified *Eps-7D* gene.

The present study is focused on understanding the interaction effect of newly identified *Eps-7D* QTL with *Ppd-D1* (the photoperiod-sensitivity gene most frequently shown to have the strongest effect; [18,19,20,21]) on phenology and related traits as well as on the initiation of primordia (leaves, spikelets and florets). The study was conducted in growth chambers under short day (12 h), otherwise the effect of *Ppd-D1* (and its interactions with *Eps-7D*) would have been unnoticed, at three temperature regimes (9, 15 and 18 °C) to evaluate to what degree the effect of *Eps-7D* and interaction with *Ppd-D1* is altered by these factors.

## 2. Results

### 2.1. Phenology

**Figure 1:**
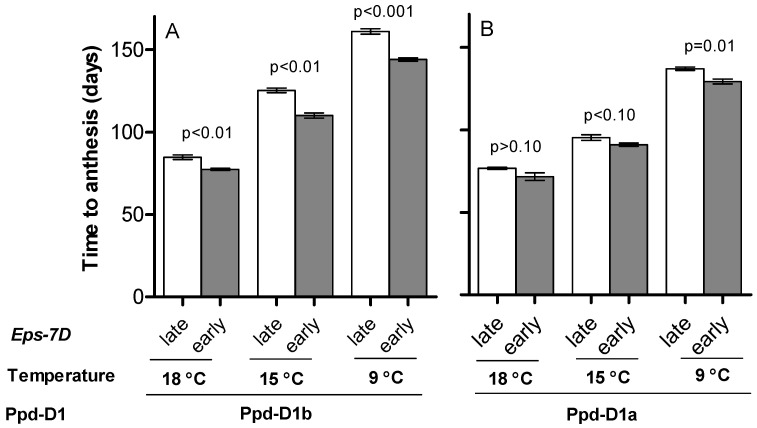
Duration of the whole phase from seedling emergence to anthesis for the lines carrying *Eps-7D-late* (open bars) or *-early* (closed bars) alleles with *Ppd-D1b* (**A**) or *Ppd-D1a* (**B**) alleles in the background under short day at three growing temperatures. Error bars indicate the SEM and the *p*-value indicates the level of significance exclusively due to the action of the *Eps-7D* gene within each temperature and *Ppd-D1* allelic condition.

Both temperature conditions (cf. different temperature regimes within the same panel of Figure 1) and the allelic status of the *Ppd-D1* gene (cf. Figure 1A,B for the same temperature regimes) affected noticeably the time to anthesis of near isogenic lines (NILs) grown under short days. Furthermore, the sensitivity of time to anthesis to temperature was affected by the photoperiod sensitivity gene in the background, particularly for the difference between 15 and 18 °C (the difference between 9 and 15 °C was similar under either of the *Ppd-D1* allele). That is, the difference in time to anthesis between 15 and 18 °C was much larger when the photoperiod allele was sensitive than when it was insensitive (cf. differences between these temperatures in Figure 1A,B). The same interaction is clear when comparing time to anthesis of lines with *Ppd-D1b* and *Ppd-D1a* alleles (cf. panels A and B of Figure 1). The difference was clearly larger at 9 and 15 °C than at 18 °C. Indeed, at 18 °C the difference between *Ppd-D1b* and *Ppd-D1a* was only 7 d (averaging across both *Eps-7D* alleles), but at that temperature, 7 d are equivalent to *ca*. 125 °C d. Therefore, sensitivity was still exhibited to the photoperiod at this high temperature, although it was less than the sensitivity at lower temperatures (at 9 °C, the difference between *Ppd-D1b* and *Ppd-D1a* lines was almost 20 d, equivalent to 175 °C d).

The effect of *Eps-7D* on time to anthesis was consistent in that the presence of *Eps-7D-late* allele, which always showed a delayed time to anthesis compared to *Eps-7D-early*, but the magnitude of these differences as well as the statistical significance clearly depended on the photoperiod sensitivity allele in the background (cf. Figure 1A,B at each temperature regime). The differences in time to anthesis between lines with the *Eps-7D-late* and -*early* alleles were larger with *Ppd-D1b* in the background (*ca*. 7, 15 and 17 d at 18, 15 and 9 °C, respectively) than with *Ppd-D1a* (*ca*. 5, 4 and 7 d at 18, 15 and 9 °C, respectively).

**Figure 2:**
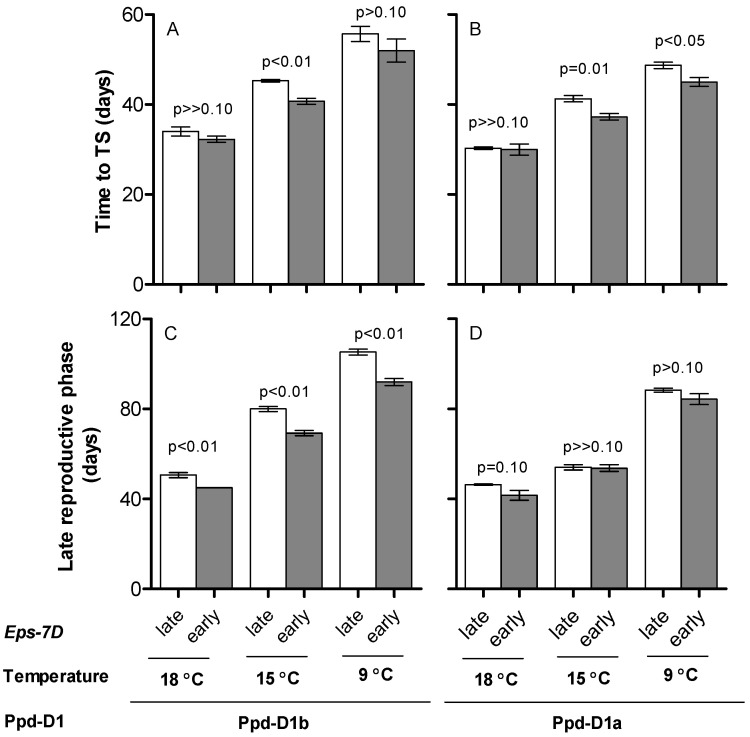
Duration of phase from seedling emergence to terminal spikelet (TS) (**A,B**) and time from then to anthesis, the late reproductive phase (**C,D**) for the lines carrying *Eps-7D-late* (open bars) or *-early* (closed bars) with *Ppd-D1b* (**A,C**) or *Ppd-D1a* (**B,D**) alleles in the background under short day at three growing temperatures. Error bars indicate the SEM and the *p*-value indicates the level of significance exclusively due to the action of the *Eps-7D* gene within each temperature and *Ppd-D1* allelic condition.

As expected, higher temperatures (cf. different temperature regimes within each of the panels of Figure 2) and the action of *Ppd-D1a* (cf. Figure 2A vs. Figure 2B and Figure 2C vs. Figure 2D for the same temperature regimes) reduced the duration of both phases.

The effect of *Eps-7D* on time to anthesis was result of a ‘domino effect’ caused by the *late* allele delaying time to TS as well as the duration of the LRP (Figure 2). Then again, the magnitude and significance of these changes was altered by the *Ppd-D1* allele in the background, the differences between lines with contrasting *Eps-7D* alleles being larger and more significant when having the *Ppd-D1b* than the *Ppd-D1a* allele in the background (Figure 2).

Which of the two phases composing time to anthesis was most affected by the action of the *Eps-7D* gene depended on the *Ppd-D1* allele in the background. When the background had the photoperiod-sensitive allele, when the effect of *Eps-7D* was largest (Figure 1), its effect on time to TS was evidently weaker than on the duration of the LRP (cf. Figure 2A,C). On the other hand, when the background had the *Ppd-D1a* allele, the overall effect of *Eps-7D* was smaller (Figure 1) and did not consistently affect any of the two component phases considered (cf. Figure 2B,D).

**Figure 3:**
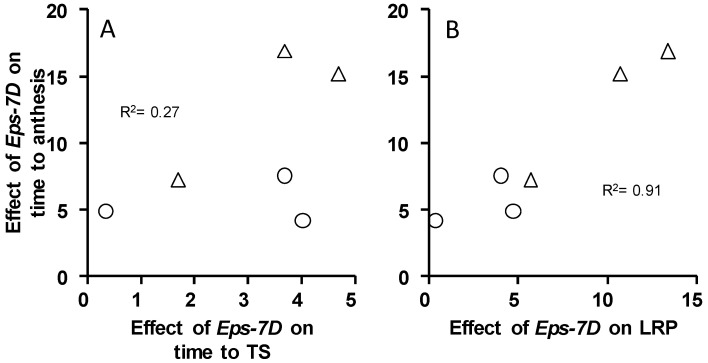
Relationships between the effects of *Eps-7D* (i.e., difference in duration between the lines with the *late* and *early* alleles) on time to anthesis and on its component phases: time from seedling emergence to terminal spikelet (TS, **A**) and time from then to anthesis, i.e., the late reproductive phase (LRP, **B**) for the lines carrying the insensitive (circles) and sensitive (triangles) *Ppd-D1* alleles in the background.

Consequently, the relationship between time to anthesis with its component phases, time from seedling emergence to TS and time from then to anthesis revealed that the *Eps-7D* effect on time to anthesis was driven by its effect on the LRP (Figure 3B) far more than by its effect on time to TS (Figure 3A). This is not only because of the proportion of the variability in the effect of *Eps-7D* on time to anthesis explained by the effects on each of the two phases, but also by the actual magnitude of the effects (note that the abscissa scale in Figure 3B is three-fold that of Figure 3A).

### 2.2. Dynamics of Leaf Appearance and Spikelet Primordia Development

As expected, FLN did not show any clear trend with temperature while the rate of leaf appearance was strongly positively affected (Table 1). On the other hand, photoperiod sensitivity (i) affected, although slightly, FLN (lines with the sensitive allele—*Ppd-D1b*—initiated on average 7.22 ± 0.17 leaves, while those carrying the insensitivity allele—*Ppd-D1a*—initiated 6.70 ± 0.14 leaves (Table 1)); and (ii) did not exhibit any clear effect on the rate of leaf appearance (averaging across temperatures and *Eps-7D* alleles, this rate was 0.089 ± 0.030 and 0.087 ± 0.026 leaves d^−1^ for lines with *Ppd-D1a* and *Ppd-D1b* alleles, respectively; Table 1).

The effect of *Eps-7D* on FLN was very subtle in that the line with the *Eps-7D-late* allele had slightly higher FLN compared to the *-early* allele (Table 1). Thus, the delayed anthesis of the *Eps-7D-late* allele was not related to the effect on FLN. Indeed, there was a consistent reduction in the rate of leaf appearance due to the action of *Eps-7D-late*, particularly when the background had the *Ppd-D1b* allele, and the reduced rate of leaf appearance was the main reason for the delayed anthesis produced by *Eps-7D-late*.

Most leaf primordia were initiated before the onset of the experiment (as *c.* four leaves are already initiated in the embryo and *c*. two more leaves can be initiated from sowing to seeding emergence). Hence, we could only have a good record of the dynamics of spikelet primordia initiation. As described above (Figure 2), there was an interaction between *Eps-7D* and *Ppd-D1* genes on the timing of TS: when the background had the sensitive *Ppd-D1b* allele, TS was delayed by the action of the *Eps-7D-late* allele, while that delay was not evident when the background had the insensitive *Ppd-D1a* allele.

**Figure 4:**
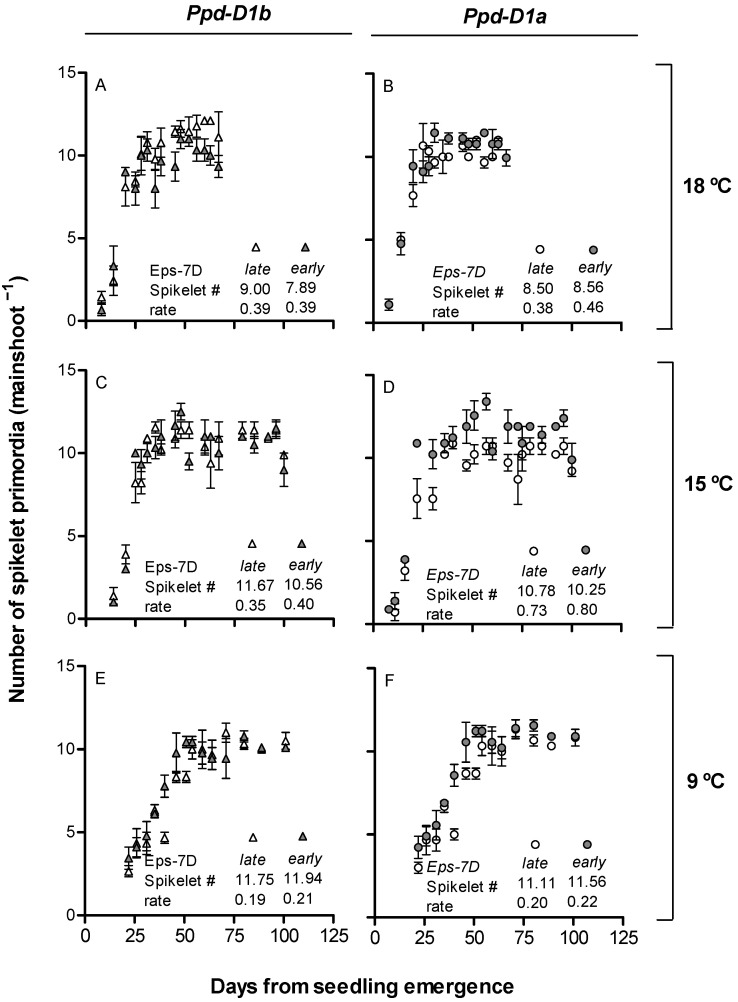
Dynamics of spikelet initiation for *Eps-7D-late* (open symbols) and *-early* (closed symbols) for the lines carrying the *Ppd-D1b* (**A**,**C**,**E**, triangles) or *Ppd-D1a* (**B**,**D**,**F**, circles) alleles in the background when grown at 18 (**A**,**B**), 15 (**C**,**D**) and 9 °C (**E**,**F**). The inset of each panel consists of the final number of spikelet primordia as well as the estimated rate of spikelet initiation (slope of the relationship until reaching TS; spikelet primordia per day).

The lines with the *Eps-7D-late* allele had *c*. one spikelet more than the line with the -*early* allele when the sensitive *Ppd-D1b* allele was present in the background under 18 and 15 °C, but not at 9 °C (Figure 4A,C,E, insets). Furthermore, the *Eps-7D* gene did not affect the final number of spikelets when the insensitive allele *Ppd-D1a* was in the background (Figure 4B,D,F, insets).

The differences in spikelet primordia produced by *Eps-7D* was mainly related to the effects on duration of spikelet initiation, as spikelets were initiated more or less at a constant rate under each temperature regime (although naturally, this rate increased with higher temperatures) (Figure 4). The effect of temperature on accelerating the rate of spikelet initiation did not compensate the effect on reducing the duration of spikelet initiation, and therefore, the total number of spikelets was higher at lower temperatures (Figure 4).

### 2.3. Spike Fertility and Dynamics of Floret Development

**Figure 5:**
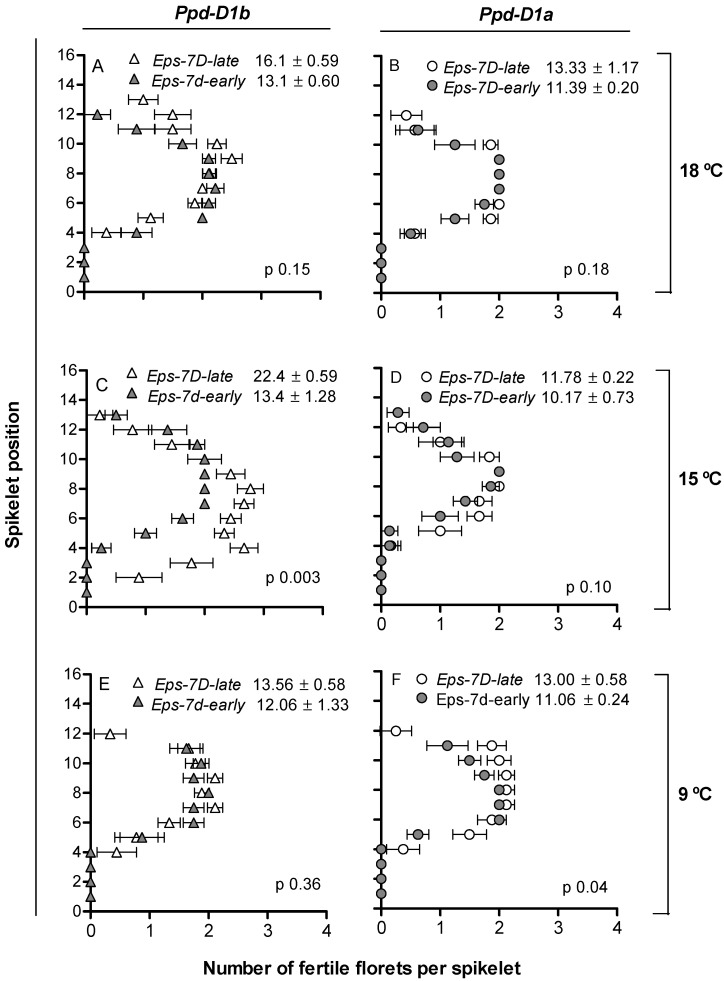
Number of fertile florets at anthesis in each spikelet along the spike (spikelet 1 is the first, most basal, spikelet) for the lines carrying *Eps-7D-late* (open symbols) or *-early* (closed symbols) allele with *Ppd-D1b* (triangles, **A**,**C**,**E**) or *Ppd-D1a* (circles, **B**,**D**,**F**) alleles in the background under short day at three growing temperatures: 18 (**A**,**B**), 15 (**C**,**D**) and 9 °C (**E**,**F**). Error bars indicate the SEM. The inset of each panel consists of the fertile florets per spike and the *p*-value indicates the level of significance of the difference between *Eps-7D-late* and *-early* lines within each condition (*Ppd-D1* in the background and growing temperature).

Spike fertility, measured as number of fertile florets per spike, was slightly yet noticeably affected by *Eps-7D* and the magnitude of the effect depended upon the *Ppd-D1* allele in the background and temperature regime. Lines with the *Eps-7D-late* allele tended to have more fertile florets per spike than that of *Eps-7D-early* across all conditions (Figure 5), but the magnitude and significance of these differences depended upon the *Ppd-D1* allele in the background and the temperature (Figure 5).

Thus, in general, the effect of the *Eps-7D* gene was more noticeable when the photoperiod-sensitive allele was in the background (Figure 5, left panels), and that difference was largest (and significant) at 15 °C, smaller and insignificant at 18 °C and even smaller at 9 °C (Figure 5, left panels).

As expected, beyond the effect of *Eps-7D*, lines with *Ppd-D1b* produced more fertile florets per spike than those with *Ppd-D1a*, although interacting with temperature as the sensitivity to photoperiod affected spike fertility at 15 and 18 °C, but not at 9 °C (Figure 5).

**Figure 6:**
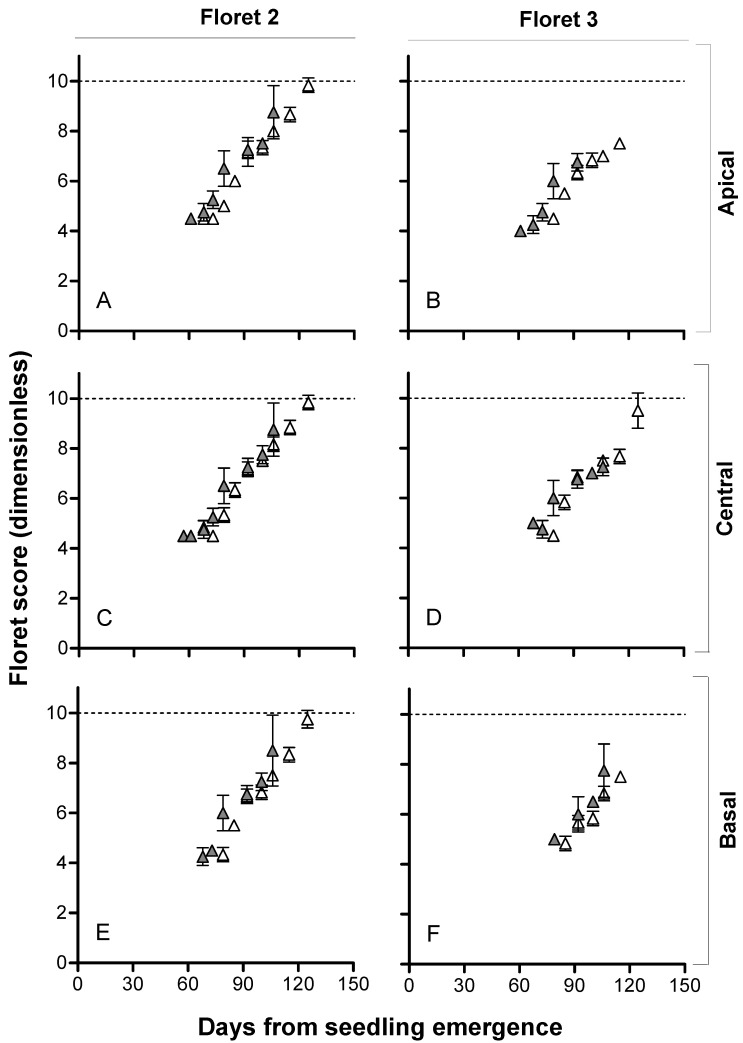
Relationship between floret development as the floret score (dimensionless) and days from seedling emergence for *Eps-7D-late* (open symbols) and -*early* (closed symbols) with sensitive allele for *Ppd-D1* (*Ppd-D1b*) in the background at 15 °C for F2 (**A**,**C**,**E**) and F3 (**B**,**D**,**F**) in spikelets from apical (**A**,**B**), central (**C**,**D**) and basal (**E**,**F**) positions of the spike. The error bars are SEMs from floret scores of replicated plants (not visible when smaller than the symbol size).

The differences in floret fertility are the final outcome of those in the dynamics of floret development. Floret 1 (i.e., the first floret developing in each spikelet and the one closest to the rachis; F1) reached stage 10 (fertile floret stage) in all lines, irrespective of the *Eps-7D* allele, the sensitivity to photoperiod given by the allele of *Ppd-D1* in the background, and the temperature regimes (data not shown, as this floret is not responsible for differences in spike fertility). As the differential developmental rates are the bases for the higher spike fertility in the *Eps-7D-late* lines when grown at 15 °C with *Ppd-D1b* in the background (Figure 5), we showed the dynamics of floret development for F2 and F3 in each of the three spikelets (apical, central and basal) considered individually (Figure 6). To broadly illustrate the effects of the treatments (Eps-7D, Ppd-D1 and temperature) on the dynamics of floret development, a supplementary figure (Figure S1) is available where we averaged the stages of development of these two floret positions (F2 and F3) of basal (third spikelet from the base), central (the middle spikelet) and apical (third spikelet from the TS) spikelets at each sampling time.

The fertility of the second floret primordium (F2) depended on the *Ppd-D1* allele in the background and temperature regime (Figure 6; and Supplementary Figure S1). The lines with *Eps-7D-late* tended to have delayed initiation of floret primordia compared to those with *Eps-7D-early*, and that effect was clearer when the background had a sensitive *Ppd-D1b* allele (Figure 6; and Supplementary Figure S1). However, as the *Eps-7D-late* also reached anthesis later, the delayed onset of floret development did not reduce the period of floret initiation (Figure 6; and Supplementary Figure S1). F2 developed until well-advanced stages, but did not always reach the stage of fertile floret (floret score 10), while in the background, the *Ppd-D1* was insensitive (Supplementary Figure S1B,F,J); in this condition (*Ppd-D1a*), there were no noticeable differences between lines carrying the *Eps-7D-late* or *-early* allele.

With *Ppd-D1b* in the background, F2 did reach W10 in all three spikelets (at least in 67% of the plants) considered under 18 and 9 °C (Supplementary Figure S1A,I, respectively) in lines carrying either of the *Eps-7D* alleles; however, at 15 °C, there was a clear difference in developmental progress of F2 between *Eps-7D-late* and *-early* lines (Supplementary Figure S1E). F3 died before reaching the stage of fertile floret in almost all cases (Supplementary Figure S1C,D,G,H,K,L), but did show a clear difference between lines with contrasting *Eps-7D* alleles mainly at 15 °C and with *Ppd-D1b* was in the background (Supplementary Figure S1G).

Although for the average of the three spikelets it is not possible to appreciate clear differences, for this particular case of lines, having *Ppd-D1b* in the background and grown at 15 °C (in which the differences in spike fertility between the contrasting *Eps-7D* lines were largest; Figure 5C), we offered the rates of floret development of florets 2 and 3 at each spikelet individually (Figure 6).

Floret 2 in *Eps-7D-late* lines when grown at 15 °C and with *Ppd-D1b* in the background clearly developed more than *Eps-7D-early* lines in all three spikelet positions considered (Figure 6, left panels). In all spikelets, F2 reached the stage of fertile florets in all or almost all plants dissected when lines were *Eps-7D-late*, while only a minor proportion of them developed to stage 10 (of fertile florets) in *Eps-7D-early* lines (Figure 6, left panels).

The third floret from the rachis aborted in the basal and apical spikelets of lines with either *Eps-7D-late* or *-early* allele, although lines *Eps-7D-late* allele did reach later stages of development in the apical spikelets (Figure 6B). On the other hand, in the central spikelets, the differences between *Eps-7D-late* and *-early* lines became clear: whilst F3 in this spikelet finally aborted in all plants sampled and floret 3 was never fertile in *Eps-7D-early* lines, most plants of the lines carrying the late allele showed F3 reaching the stage of fertile floret (Figure 6D).

## 3. Discussion

The aim of this work was to study the *Eps-7D* × *Ppd-D1* interaction under contrasting temperatures to evaluate to what degree the effects of the newly identified *Eps-7D* depended upon the photoperiod sensitivity in the genetic background. Naturally, we also obtained results on the effects of *Ppd-D1* and the temperature on the processes studied. All these effects, occasionally commented in the description of the results, were commensurate with what is well known in the literature [2,8,10,21,22,23,24,25,26,27,28,29,30,31,]. Thus, we will concentrate this discussion on the aim of the study: the effects of this new *Eps-7D* gene as well as the dependence on the sensitivity to photoperiod for having these effects.

A clear interaction between *Eps-7D* and *Ppd-D1* was evidenced, not only on the phenology (e.g., the delay in time to anthesis due to the *late* allele of *Eps-7D* in presence of sensitive *Ppd-D1b* was much stronger compared to that produced by the same allele on *Ppd-D1a*) but also in terms of rates of leaf appearance and of floret development. Some of these *Eps-7D* × *Ppd-D1* interactions were evident at all temperatures considered (e.g., phenology), while others were clear only at particular temperatures (e.g., floret development and the resulting effects of *Eps-7D* on spike fertility). Interactions between *Eps* and *Ppd* genes were identified before [13,17] and could well be a major reason for the well acknowledged *Eps* × genetic background interaction [32,33]. This interaction is relevant as it means that one or other *Eps* gene could potentially be exploited in lines with contrasting sensitivity to the photoperiod. In this particular case, to exploit the effects of this new *Eps-7D* gene, it should be introgressed in lines that are highly sensitive to the photoperiod.

The fact that the *Eps-7D* × *Ppd-D1* interactions would be clearer at some temperatures as opposed to others is also something that could have been expected as both *Eps-7D* [1], as well as other *Eps* [7,9,14,15,34,35], and *Ppd* genes [12,36,37] have shown to interact with temperature.

In the background of *Ppd-D1b*, in which the effects of *Eps-7D* were far more relevant, it was clear that this *Eps* gene affected phenology mainly during the LRP. This is relevant as it is during this phase that spike fertility is determined [22,38,39,40,41,42,43]. Knowledge of genetic factors affecting particular phases of wheat phenology would help in tailoring not only time to anthesis but also the partitioning of this time into particular phases.

The effect of *Eps-7D* on FLN was subtle even under sensitive *Ppd-D1* background where the *Eps-7D-late* significantly delayed phenology. This subtle effect is commensurate with the fact that *Eps-7D-late* mainly affected development during later stages, and in agreement with other *Eps* genes mainly affecting reproductive development [14,24]. Indeed, the effect of *Eps-7D-late* on the duration of the LRP (encompassing time to flag leaf emergence) was the consequence of this allele decreasing the rate at which leaves appeared, lengthening the phyllochron and consequently delaying the appearance of the flag leaf.

The lines carrying the *Eps-7D-late* tended to increase spike fertility consistently compared to those with *Eps-7D-early*, although the effect was important only when the background was photoperiod-sensitive and when the temperature was intermediate. This slight improvement in fertility at individual spikelet level was a result of extending the floret development period, which allowed labile florets (F2 and F3 in this case, depending on the particular spikelets considered) to reach the stage of fertile floret. This means that the mechanism by which the *Eps-7D-late* allele improved spike fertility was in line with what had been seen in other studies in the literature ([14,15] and references quoted therein).

This *Eps-7D* interacted with temperature [1] and *Eps-2B* [16], and the interaction effect have been shown to be effective well beyond the duration of developmental phases. Our findings suggest that it is not only important to study every newly identified *Eps* gene (simply because although they are all named equally, the mechanisms by which they actually modify time to anthesis may be quite different), but it is also necessary to evaluate their functions under varying backgrounds of other important genetic factors affecting wheat development to ensure repeatability of their effect and their application. The *Eps* QTL studied here brought about subtle changes in time to anthesis, similar to most other *Eps* studies before [4,15,24,44], which naturally had a weak consequential effect on spike fertility, although under particular circumstances (*Ppd-D1* in the background and temperature) these effects were relevant. This is important to be considered when particular *Eps* genes are to be exploited in wheat breeding.

## 4. Materials and Methods

For details, please see the companion paper [1]. To recap briefly, treatments considered in this paper comprised four NILs differing in the alleles for *Eps-7D* and *Ppd-D1* (i.e., *Eps-7D-late* + *Ppd-D1a*; *Eps-7D-late* + *Ppd-D1b; Eps-7D-early* + *Ppd-D1a*; *Eps-7D-early* + *Ppd-D1b*); grown at three temperatures (namely, 9, 15 and 18 °C) from seedling emergence to anthesis. We only considered here the plants grown under short day (12 h); otherwise, the effect of *Ppd-D1* and its interactions with *Eps-7D* would have remained unnoticed.

As detailed in the companion paper [1], we determined not only the timing of the important growth stages (seedling emergence (DC10), onset of stem elongation (DC30), flag leaf, heading (DC39), and anthesis (DC65); following the Zadoks’ scale [45]), but also periodically (i) the number of leaves appearing on the main shoot was recorded following the Haun scale [46], and with that, we described the pattern of leaf appearance, and (ii) the apex stages and number of primordia initiated—firstly analysing the apex as a whole, and secondly, after the TS stage we dissected the individual spikelets and determined the number and stage of floret primordia (following the apex scales of Kirby and Appleyard, [47] and floret score proposed by Waddington et al., [48]). Finally, at anthesis, we counted the number of fertile florets at each spikelet of the spikes (the first spikelet being the most basal and the last being the terminal spikelet) to record the total number of fertile florets per spike.

Data were analysed considering a full factorial model using JMP Pro version 14.0 (SAS Institute Inc., Cary, NC, USA) through a two-way ANOVA; in addition, one-way ANOVA was used to determine the significance of differences between pairs of NILs carrying contrasting alleles of *Eps-7D* at each combination of *Ppd-D1* allele in the background and temperature.

## Figures and Tables

**Table 1:** Final leaf number (FLN), rate of leaf appearance (RLA; estimated as the slope of the linear regression of leaf number vs. thermal time), and the coefficient of determination for that regression (R^2^), in NILs of contrasting *Eps-7D* alleles with backgrounds of contrasting *Ppd-D1* alleles at three temperatures under short day. ***: highly significant correlation.

Growing Conditions		Allele at *Eps-7D*	FLN	RLA (Leaves d^−1^)	r^2^
***Ppd-D1b***	**18 °C**	*late*	7.0 ± 0.0	0.114 ± 0.001	0.986 ***
		*early*	6.9 ± 0.1	0.123 ± 0.001	0.983 ***
	**15 °C**	*late*	7.9 ± 0.1	0.076 ± 0.001	0.981 ***
		*early*	7.6 ± 0.1	0.084 ± 0.001	0.975 ***
	**9 °C**	*late*	7.0 ± 0.0	0.061 ± 0.000	0.987 ***
		*early*	6.9 ± 0.1	0.066 ± 0.001	0.982 ***
***Ppd-D1a***	**18 °C**	*late*	7.0 ± 0.0	0.126 ± 0.001	0.983 ***
		*early*	6.6 ± 0.2	0.130 ± 0.002	0.975 ***
	**15 °C**	*late*	7.0 ± 0.0	0.067 ± 0.001	0.980 ***
		*early*	6.8 ± 0.0	0.072 ± 0.001	0.973 ***
	**9 °C**	*late*	6.7 ± 0.2	0.067 ± 0.001	0.962 ***
		*early*	6.1 ± 0.1	0.071 ± 0.001	0.976 ***

*** All linear regressions were highly significant (*p* < 0.001).

## Data Availability

Data are available upon request from the corresponding authors.

## Supplementary Materials

The following are available online https://www.mdpi.com/2223-7747/10/3/533/s1, Figure S1. Relationship between floret development as floret score (dimensionless) and days from seedling emergence for *Eps-7D-late* (open symbols) and -*early* (closed symbols) with *Ppd-D1b* (A,C,E,G,I,K) and *Ppd-D1a* allele (B,D,F,H,J,L) in the background at 18 (A–D), 15 (E–H) and 9 °C (I–L) for F2 (left panels: A,B,E,F,I,J) and F3 (right panels: C,D,G,H,K,L). The error bars are standard error of the mean from the floret score of florets from apical, central and basal spikelet.
