# Supplementary material for: Phenology and Floret Development as Affected by the Interaction between Eps-7D and Ppd-D1
Source: Plants (Basel). 2021 Mar 12;10(3):533. doi: 10.3390/plants10030533 (PMC8001856; doi:10.3390/plants10030533)
Supplement: Supplementary file 1 [file plants-10-00533-s001.pdf]

Phenology and floret development as affected by the interaction between *Eps-7D* and *Ppd-D1*

PA Basavaraddi, R Savin, S Bencivenga, S Griffiths, GA Slafer

Supplementary file

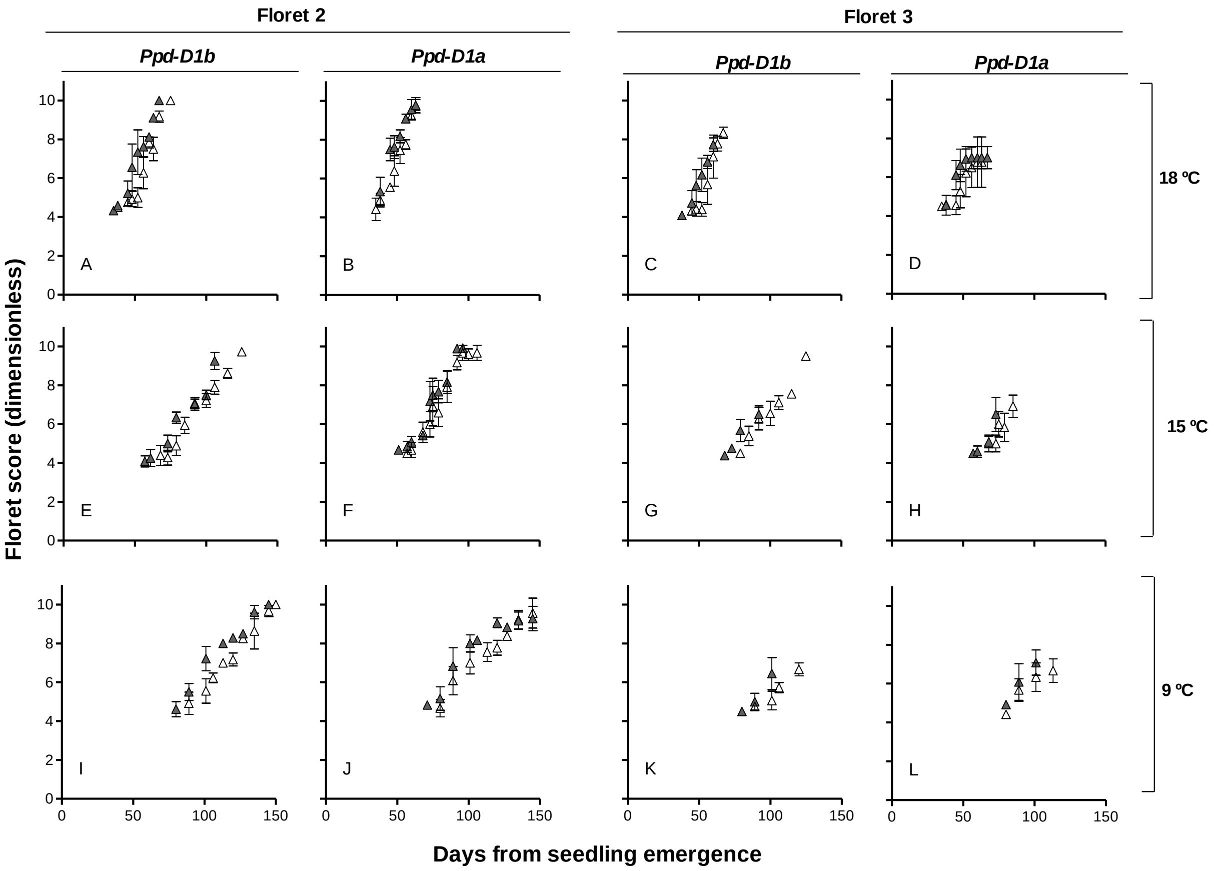

Supplementary Figure S1. Relationship between floret development as floret score (dimensionless) and days from seedling emergence for *Eps-7D-late* (open symbols) and -*early* (closed symbols) with *Ppd-D1b* (A,C,E,G,I,K) and *Ppd-D1a* allele (B,D,F,H,J,L) in the background at 18 (A-D), 15 (E-H) and 9 ºC (I-L) for F2 (left panels: A,B,E,F,I,J) and F3 (right panels: C,D,G,H,K,L). The error bars are standard error of mean from floret score of florets from apical, central and basal spikelet.
